# Causes of kidney failure among patients undergoing maintenance hemodialysis in Somalia: a multi-center study

**DOI:** 10.1186/s12882-023-03402-z

**Published:** 2023-11-27

**Authors:** Hamze Ibrahim Rage, Suleyman A Ers, Abdirazak Y Kahin, Muraad M Elmi, Abdiaziz A Mohamed, Pranaw Kumar Jha

**Affiliations:** 1 Department of Nephrology, Hargeisa Group hospital, Hargeisa, Somaliland; 2 Sheikh Khalifa Bin Zayed Al-Nahyan Hospital, Burao, Somaliland; 3 Bosaso general Hospital, Puntland, Somalia; 4 Department of Nephrology, Medanta – The Medicity, Gurugram, Haryana India

**Keywords:** Somalia, Kidney failure, Haemodialysis, Hypertension, Diabetes, Chronic kidney disease

## Abstract

**Background:**

Kidney failure is one of the leading causes of morbidity and mortality worldwide. The incidence of kidney failure in Somalia has been increasing in recent years. There is no data available on the causes of chronic kidney disease (CKD) leading to kidney failure in Somalia.

**Methods:**

This is a multicentre, descriptive cross-sectional study designed to determine the aetiology of kidney failure among patients receiving haemodialysis in four major demographic areas of Somalia. The study was conducted over a one-year period, from June 2021 to June 2022. Participants were eligible for inclusion if they had been diagnosed with kidney failure, were on regular haemodialysis, and were over 18 years of age.

**Results:**

A total of 127 patients were evaluated, 84 (66.1%) were males and 43 (33.9%) were female. The mean age of kidney failure patients was 49.3 ± 12.2 years. They originated from various regions, 5.6% from the south, 29.9% from the north-eastern, and 64.5% from the northwest. The mean duration of haemodialysis was 4.4 ± 2.2 years. The most common cause of kidney failure in our study was hypertension (33.1%), followed by diabetes mellitus (27.6%), uncertain aetiology (24.4%), glomerulonephritis (7.1%), obstructive uropathy (3.8%), renovascular hypertension (1.6%), neurogenic bladder, polycystic kidney disease, congenital and hereditary diseases (0.8%).

**Conclusions:**

Our study showed the leading cause of kidney failure among maintenance haemodialysis patients was hypertension, followed by diabetes mellitus. To reduce the burden of kidney failure in Somalia, primary prevention of hypertension and diabetes and early detection and prompt management of chronic kidney disease (CKD) in high-risk populations should be a fundamental focus.

## Introduction

Chronic kidney disease is a global health concern. Kidney Disease Improving Global Outcome (KDIGO) defined CKD as an abnormality of kidney function or structure with an eGFR < 60 mL/min/1.73 m2 for at least 3 months [1]. Many patients with CKD progress to kidney failure, which requires permanent renal replacement therapy or kidney transplantation [2]. Geographical differences exist in the prevalence of CKD [3]. The prevalence of CKD is estimated to be around 13.9% in Sub-Saharan Africa [4]. There is a paucity of data available on the cause of CKD in patients with kidney failure who are on dialysis in Somalia. Due to a combination of wars and poverty, Somalia’s healthcare facilities are underdeveloped, and the majority of the population lacks access to basic services. The country’s overall morbidity and mortality remain very high, and it has the lowest health and well-being indicators globally [5]. The main risk factors for CKD in both developed and developing countries are hypertension and diabetes mellitus [6]. As previously observed, the burden of end-stage renal disease (ESRD) is exacerbated by extreme poverty [7, 8]. Kidney function can rapidly deteriorate to kidney failure in the absence of early detection and appropriate management of CKD [9]. Providing care before the onset of kidney failure is essential in mitigating the progression to kidney failure and the resulting mortality [10, 11]. In Somalia, similar to many other African countries, premature mortality significantly impacts the majority of individuals suffering from kidney failure. [12].

Renal replacement therapy was introduced in Somalia in 2012. The causes of kidney failure in Somalia among patients receiving haemodialysis are still not available. The aim of this study is to report the causes of kidney failure in major cities in Somalia among the prevalent dialysis population. This information will be of significance to the health authorities in developing and implementing appropriate kidney care services.

## Methods

### Study settings and design

We conducted a multicentre, cross-sectional study in four public dialysis centres in Somalia to determine the aetiology of kidney failure in patients on regular haemodialysis. The study was conducted from June 2021 to June 2022.

### Inclusion and exclusion criteria

Participants were eligible for inclusion if they were diagnosed with kidney failure, were on regular haemodialysis, and were over 18 years old. Patients with known acute kidney injury (AKI) on dialysis, under 18 years of old, incomplete medical records, and failed kidney transplant on dialysis were excluded from the study.

### Data collection

After obtaining written informed consent, socio-demographic variables were recorded and the patient’s medical files were reviewed to obtain other information (e.g., duration of haemodialysis, diabetes mellitus, hypertension). The medical history was recorded through a direct interview administered by a trained staff member. The final diagnosis of the causes of kidney failure was obtained from the reports of kidney biopsies if available and radiological tools (such as CT scans, ultrasound, etc.). Diabetic nephropathy was diagnosed based on a history of diabetes mellitus, hypertension, and the presence of diabetic retinopathy. On the other hand, hypertensive nephropathy was diagnosed as the cause based on a long history of hypertension that predates kidney dysfunction, accompanied by proteinuria of less than 2 g per day, the presence of left ventricular hypertrophy, and an absence or lack of evidence of other kidney diseases [13]. Polycystic kidney disease, reno-vascular conditions and obstructive uropathy, neurogenic bladder were identified through a combination of abdominopelvic ultrasound, CT scan, magnetic resonance angiogram, cystoscopy and other relevant radiological reports. Glomerulonephritis was diagnosed by referring to past kidney biopsy reports. All the collected data was entered into a Microsoft Excel spreadsheet after being double-checked; with duplicates and incomplete entries meticulously excluded.

The study was carried out in accordance with relevant guidelines and regulations, and it received approval from the administrative review boards of Hargeisa Group Hospital (Ref: HGH/16/543), and Bosaso General Hospital (Ref: BGH/0746/22).

### Statistics

The data was analysed using IBM SPSS 25. Results for continuous variables were reported as either mean values ± standard deviation or median with range. Categorical variables were presented as percentages. Tables and charts were used to present the data.

## Results

A total of 127 kidney failure patients on regular haemodialysis were included in the study. The demographic characteristics of the study participants are shown in Table 1.

The causes of kidney failure are shown in Table 2. The most common cause of kidney failure in our participants was hypertension (33.1%), followed by diabetes mellitus (27.6%), cases with uncertain or unknown aetiology (24.4%), and glomerulonephritis (7.1%). A smaller portion of enrolled patients had kidney failure attributed to obstructive-uropathy (3.8%), and renovascular (1.6%). Neurogenic bladder, polycystic kidney disease, and congenital conditions were observed in 0.8% of cases each. In terms of additional parameters, 7.9% of patients were found to have HBV, while 3.1% of patients tested positive for HCV. Regarding the cost of hemodialysis treatment, it was fully covered for 90.6% of the patients. For the remaining 9.4%, some of the costs were partially borne by the patients in the form of administrative fees and for certain supplies.

The results of regional differences in the causes of kidney failure in Somalia are shown in Table 3. Regional variations in the causes of kidney failure in Somalia were found. The Northwest Somalia had 34.1% DM, 28% hypertension, and 21.9% unknown causes as the aetiology of kidney failure. In north-eastern Somalia, 18.9% had DM, 43.2% had hypertension, and 29.7% had an unknown cause as the aetiology. A small number of patients (6.3%) were from south Somalia, where hypertension was the most common cause (37.5%).

Cause of kidney failure in different age groups is shown in Table 4. In the age group between 51 and 70 years the major cause was hypertension (50%) followed by diabetes mellitus (28%). In patients of the age group between 30 and 50 years, unknown aetiology was the predominant cause (32.3%) followed by diabetes mellitus (27.7%) and hypertension (21.5%).

**Table 1:** Demographics and baseline characteristics of the study participants

Baseline Characteristics	Value (N = 127)
**Age** (Mean ± SD) in years	49.3 ± 12.2
**Duration of Haemodialysis in years**	4.4 ± 2.2
**Gender**	
Male Female	84 (66.1%) 43 (33.9%)
**Occupation**	
Employed Unemployed	49 (38.6%) 78 (61.4%)
**Type of vascular access**	
Central venous catheter Arteriovenous fistula	43 (33.9%) 84 (66.1%)
**Hepatitis**	
HBV HCV	10 (7.9%) 4 (3.1%)
**Origin**	
Northwest Somalia Northeast Somalia South Somalia	82 (64.5%) 37 (29.1%) 8 (6.3%)
**HD treatment cost**	
Paid Free	12 (9.4%) 115 (90.6%)

**Table 2:** Etiology of kidney failure among patients undergoing hemodialysis

Variables	Frequency(n)	Percentage%
Hypertension	42	33.1%
Diabetes Mellitus	35	27.6%
Unknown/aetiology uncertain	31	24.4%
Glomerulonephritis	9	7.1%
Obstructive uropathy	5	3.8%
Reno-Vascular hypertension	2	1.6%
Neurogenic bladder	1	0.8%
Polycystic kidney disease	1	0.8%
Congenital (hereditary diseases)	1	0.8%
Total	127	100.0%

**Table 3:** Regional differences in causes of kidney failure

Cause of KF	Total		South	Northeast	Northwest
Hypertension		42	3(37.5%)	16 (43.2%)	23 (28%)
Diabetes Mellitus		35	0 (0%)	7 (18.9%)	28 (34.1%)
Unknown		31	2 (25%)	11 (29.7%)	18 (21.9%)
Glomerulonephritis		9	2 (25%)	1 (2.7%)	6 (7.3%)
Obstructive uropathy		5	0 (0%)	0 (0%)	5 (6.1%)
Neurogenic bladder		1	1 (12.5%)	0 (0%)	0 (0%)
Congenital		1	0 (0%)	1 (2.7%)	0 (0%)
RVH		2	0 (0%)	1 (2.7%)	1 (1.2%)
PKD		1	0 (0%)	0 (0%)	1 (1.2%)
**Total**		127 (100%)	8 (6.3) %	37 (29.1%)	82 (64.5%)

KF = kidney failure; RVH = renovascular hypertension; PKD = polycystic kidney disease

**Table 4:** Aetiology of kidney failure in different age groups

Causes of kidney failure	Age Groups (y)				Total
	< 29	30–50	51–70	> 71	
Hypertension	0 (0%)	14 (21.5%)	25 (50%)	3 (60%)	42 (33.1%)
Diabetes Mellitus	2 (28.6%)	18 (27.7%)	14 (28%)	1 (20%)	35 (27.6%)
Unknown	3 (42.8%)	21 (32.3%)	7 (14%)	0 (0%)	31 (24.4%)
Glomerulonephritis	2 (28.6%)	5 (7.7%)	2 (4%)	0 (0%)	9 (7.1%)
Obstructive-uropathy	0 (0%)	2 (3.1%)	2 (4%)	1 (20%)	5 (3.8%)
Neurogenic bladder	0 (0%)	1 (1.5%)	0 (0%)	0 (0%)	1 (0.8%)
Congenital	0 (0%)	1 (1.5%)	0 (0%)	0 (0%)	1 (0.8%)
RVH	0 (0%)	2 (3.1%)	0 (0%)	0 (0%)	2 (1.6%)
PKD	0 (0%)	1 (1.5%)	0 (0%)	0 (0%)	1 (0.8%)
**Total**	**7**	**65**	**50**	**5**	**127**

RVH = renovascular hypertension; PKD = polycystic kidney disease

## Discussions

The precise prevalence of kidney failure in Somalia remains unknown; however, it is presumed to be notably high. In contrast to most developed nations, Somalia has not yet established a comprehensive national renal registry. As a result, there is a dearth of precise data regarding the prevalence of kidney diseases within the country. There is a limited availability of renal replacement therapy modalities in Somalia. Unfortunately, the current infrastructure does not support the provision of peritoneal dialysis or kidney transplantation services. Therefore, haemodialysis remains the sole viable alternative. The haemodialysis centres in the southern region of Somalia predominantly operate within the private sector, with patients bearing the financial responsibility for their treatment. Haemodialysis centres can be found in public regional hospitals within Somaliland, a de facto state. These centres offer dialysis services at no cost to patients. In the Puntland region, one publicly accessible dialysis facility is currently available at no cost to patients. However, it is important to note that certain supplies related to dialysis procedures may require payment from the patient. Unfortunately, a significant proportion of patients in Somalia face significant challenges in accessing dialysis services owing to limited availability. Currently, these services are primarily concentrated in major urban areas, resulting in a scarcity of dialysis centres throughout the country. The average cost per high-definition (HD) session in Somalia ranges from 35 to 65 USD, which is comparable to the rates observed in neighboring countries. [14, 15]. The total unemployment rate among the patients was 61.7%. Previous studies have shown that many kidney failure patients are unemployed [16].

Our study found that the mean age of kidney failure patients was 49.3 years, and that hypertensive kidney disease (33.1%) was the most common cause of kidney failure, followed by diabetes mellitus (27.6%). A recent study from a tertiary care hospital in Mogadishu found that, diabetes accounted for 39.4% of kidney failure cases, followed by hypertension (35.6%) [17]. A Systematic Review and Meta-Analysis, found that the prevalence of chronic kidney disease (CKD) among patients with hypertension in sub-Saharan Africa was 17.8% (95% CI 13.0–23.3%, I2 = 95.5% [19]. A study from Ethiopia found that 225 (51.6%) of kidney failure patients receiving haemodialysis had hypertension as their main cause of CKD, followed by 130 (29.8%) diabetes and 137(31.4%) glomerulonephritis [19]. In Uganda, the prevalence of CKD in the community varies between 2 and 7% and up to 15% among patients with HIV or hypertension [20–22]. Establishing a clinical diagnosis of hypertensive nephropathy can be challenging because hypertension can exacerbate the progression of any underlying renal insufficiency, and kidney disease itself can lead to secondary hypertension [23]. We conducted a review of clinical documentation related to end-stage renal disease attributed to hypertension, [24]. employing the following criteria: a prolonged history of hypertension that predated kidney dysfunction, proteinuria of less than 2 g per day, the presence of left ventricular hypertrophy and the absence or lack of evidence of other kidney diseases [25]. Similar criteria were used among haemodialysis patients previously [26]. Hypertension linked to kidney diseases poses a more significant challenge among Black individuals when compared to other ethnicities, and a substantial part of this inequality can be attributed to genetic variations in the APOL1 gene. [27].

Our study found that majority of kidney failure patients under the age of 50 had an unknown aetiology. This is likely due to the unavailability of kidney biopsy in Somalia. In sub-Saharan Africa (SSA), the primary causes of chronic kidney disease (CKD) differ from those in the rest of the world, with glomerulonephritis and infections, such as HIV/AIDS, accounting for high percentage of cases [28, 29]. In our study, the unknown cause accounted for 24.4% of kidney failure cases, while glomerulonephritis was the cause in 7.1% of cases, where a proven diagnosis of GN was made based on kidney biopsy reports. Glomerular diseases are common in Africa and are a major cause of kidney failure, but remains poorly characterized due to lower renal biopsy rates [30]. In Nigeria, a study among haemodialysis patients reported that hypertension and chronic glomerulonephritis were the most common causes of kidney failure [31]. In our study, the prevalence of hepatitis B virus (HBV) infection was 7.9%, and the prevalence of hepatitis C virus (HCV) infection was 3.1%. These findings are similar to those of a recent study in Somalia, which reported a prevalence of HBV of 7.3% and a prevalence of HCV of 3.2% among haemodialysis patients [32].

With the increasing prevalence of diabetes mellitus (DM) worldwide, it has become the primary driver of chronic kidney disease (CKD) and kidney failure in developed nations, and developing countries are quickly following this pattern. [33]. In our findings, the second leading cause of kidney failure in Somalia was diabetes mellitus, seen in 27.6% of patients. Risk factors for diabetic kidney disease include advancing age, male gender, long-term diabetes, smoking, obesity, high blood pressure, and genetic susceptibility [34]. Modifiable risk factors such as, smoking, chewing chat(Qat), physical inactivity and obesity tend to have an effect in this region. Many patients in Somalia are undiagnosed or under-recognized at early stages until the disease has progressed to an advanced state. The Hargeisa Group Hospital Dialysis Unit is witnessing a steady increase in the number of kidney failure patients, and a significant majority of these individuals clearly require the immediate initiation of Renal Replacement Therapy (RRT). Misconception about dialysis is a huge contributor to mortality among HD patients in Somalia. Lack of knowledge and awareness are some of the main contributing factors to the progression of CKD, leading to kidney failure.

### Strengths and limitations

Our study has several important limitations. First, it only includes patients on maintenance haemodialysis at four participating centres,, which may not be representative of the entire population. Second, the diagnosis of hypertensive nephropathy has limitations as mentioned previously, and not all the causes of kidney failure in our study were biopsy proven. Third, referral bias is possible, as patients may have been referred to the participating centres based on their socioeconomic status, which could lead to over-representation of certain causes of kidney failure in different populations. The strength of this study is that it included of all kidney failure patients receiving haemodialysis treatment in four major demographic cities with an estimated 3.4 million population residents. This is the first multicentre study in Somalia to describe the causes of kidney failure among patients on regular haemodialysis.

## Conclusions

Our study showed that hypertension was the leading cause of kidney failure among haemodialysis patients in Somalia, followed by diabetes mellitus. To reduce the burden of kidney failure in Somalia, primary prevention of hypertension and diabetes, as well as appropriate and timely treatment of chronic kidney disease (CKD), is essential. The development and implementation of a stronger healthcare system and comprehensive, well-coordinated chronic kidney disease (CKD) prevention programs are essential to improving kidney health and reducing the burden of kidney disease.

## Data Availability

All data generated or analysed during this study are included in this published article.

## Abbreviations

AKI: Acute kidney injury
APOL1: Apolipoprotein L1
CKD: Chronic kidney disease
ESRD: End stage renal disease
SSA: Sub-Saharan Africa
HD: Haemodialysis
HBV: Hepatitis b virus
HCV: Hepatitis b virus
GN: Glomerulonephritis
PKD: Polycystic kidney disease
DM: diabetes
RVH: Renovascular hypertension
RRT: Renal replacement therapy
KDIGO: Kidney Disease Improving Global Outcome
HIV/AIDs: Human immunodeficiency virus/Acquired immunodeficiency syndrome
